# The complete chloroplast genome of *Mucuna sempervirens* (Fabaceae: Papilionoideae)

**DOI:** 10.1080/23802359.2021.1914232

**Published:** 2021-06-07

**Authors:** Li Chen, Yan-Meng Huang, Ying-Ying Qian, Zhi-Bing Wan

**Affiliations:** College of Life and Environment Sciences, Huangshan University, Huangshan, China

**Keywords:** Chloroplast genome, *Mucuna sempervirens*, phylogenetic analysis

## Abstract

The complete chloroplast genome of *Mucuna sempervirens* reported herein was a circular DNA molecule of 154,542 bp in length. The genome had a typical quadripartite structure, consisting of a pair of inverted repeats (IRa and IRb: 24,836 bp) separated by a large single-copy region (LSC: 67,996 bp) and a small single-copy region (SSC: 18,363 bp). The overall GC content of the genome was 35.1%. The cp genome encoded a set of 128 genes, containing 82 protein-coding genes, 37 tRNA genes, and eight rRNA genes. Phylogenetic analysis indicated that *M*. *sempervirens* was sister to *M*. *macrocarpa*. These findings may provide useful information to the phylogeny of the genus *Mucuna*.

*Mucuna sempervirens* Hemsl is a lianas evergreen species belong to the genus *Mucuna* (Fabaceae: Papilionoideae). They grow in subtropical forests, bushes, valleys, and rivers, which are widely distributed in northwest China (Sichuan, Guizhou, Yunnan, Shaanxi, Hubei, Zhejiang, Jiangxi, Hunan, Fujian, Guangdong, and Guangxi), and Japan. It has a high value of ornamental and medicinal application (Du and Li 2012).

Fresh leaves of *M*. *sempervirens* were sampled from Lianfeng peak, Jiuhuashan Mountain, Anhui Province, China (30.51 N, 117.85E, altitude 236 m). The voucher specimen was preserved and deposited in the Museum of Huangshan University (Voucher number: YMT2021; Contact person: ZB Wan and email: wanzb626@hsu.edu.cn). Total DNA extraction and whole-genome sequencing were conducted by Nanjing Genepioneer Biotechnologies Inc. (Nanjing, China) with the Illumina Hiseq X Ten platform. Raw data was filtered using the fatap 0.20.0 (Chen et al. 2018). A total of 20,341,388 clean reads were accomplished by CLC v9.11 (Nicolas et al. 2017). The alignment of contigs was under the BLAST algorithm (Johnson et al. 2008) with *M*. *macrocarpa* (NC044116) plastid genome as reference. The genome was annotated using the CpGAVAS pipeline (Liu et al. 2012) and submitted to GenBank (Accession number: MW556306). MsatCommander v0.8.2.0 (Faircloth, 2008) was utilized to identify simple sequence repeats (SSRs).

The complete cp genome of *M*. *sempervirens* was 154,542 bp in length and exhibited a typical quadripartite structure, which was composed of a pair of inverted repeats (IRa and IRb: 24,836 bp) separated by a large single-copy region (LSC: 67,996 bp) and a small single-copy region (SSC: 18,363 bp). The overall GC content of the genome was 35.1%, while the corresponding values of LSC, SSC, and IR regions were 32.9%, 30.5%, and 42.3%, respectively. A total of 128 genes were annotated in the cp genome, including 82 protein-coding genes, 37 tRNA genes, and eight rRNA genes.

To investigate phylogenetic position of *M*. *sempervirens*, the sequence alignment was performed on the 24 cp genome sequences using MAFFT 7.307 (Katoh et al. 2019), and then jModelTest v2.1.7 (Darriba et al. 2012) was utilized to decide the optimal model, including 22 Phaseoleae species and 2 Ormosieae species as outgroup. Maximum likelihood (ML) method was used to reconstruct phylogenetic tree by online RAxML BlackBox software in http://www.phylo.org (Stamatakis et al. 2008). The phylogenetic analysis result shows that *M*. *sempervirens* is sister to *M*. *macrocarpa* and resides within the clade representing the genus *Mucuna* (Figure 1) with highly bootstrap support values. It indicated that our new determined complete cp genome sequence could meet the demands and explain some evolution issues.

**Figure 1.**
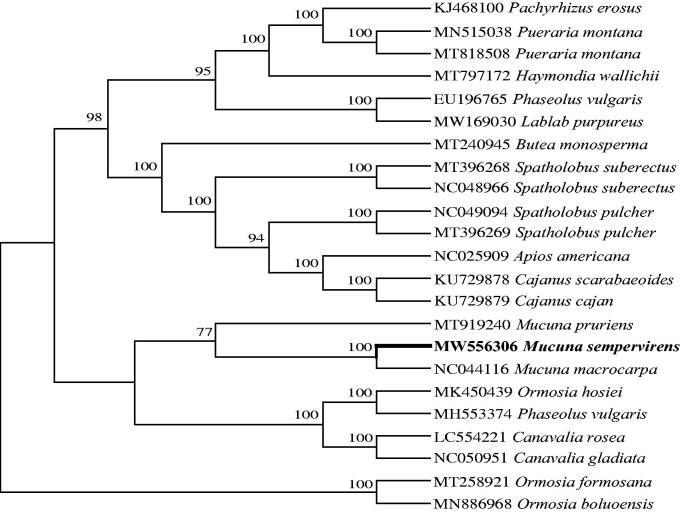
Maximum likelihood (ML) tree based on chloroplast genome sequences of 21 Phaseoleae species with 2 Ormosieae species as outgroups. Values above branches correspond to ML bootstrap percentages.

## Data Availability

The complete chloroplast genome sequence and annotation of *M*. *sempervirens* that support the findings of this study are openly available in GenBank of NCBI at [https://www.ncbi.nlm.nih.gov] under the accession no. MW556306.
